# Targeting C-Type Lectin Receptors for Cancer Immunity

**DOI:** 10.3389/fimmu.2015.00408

**Published:** 2015-08-24

**Authors:** Huimin Yan, Tomomori Kamiya, Papawee Suabjakyong, Noriko M. Tsuji

**Affiliations:** ^1^Immune Homeostasis Laboratory, Biomedical Research Institute, National Institute for Advanced Industrial Science and Technology (AIST), Tsukuba, Japan; ^2^Institute for Liver Disease, Fifth Hospital of Shijiazhuang, Shijiazhuang, China; ^3^Research Institute for Biomedical Sciences, Tokyo University of Science, Noda-shi, Japan; ^4^Department of Clinical and Analytical Biochemistry, Graduate School of Pharmaceutical Sciences, Chiba University, Chiba-shi, Japan

**Keywords:** C-type lectin receptors, innate immunity, cancer immunity, immunoregulation

## Abstract

C-type lectin receptors (CLRs) are a large family of soluble and trans-membrane pattern recognition receptors that are widely and primarily expressed on myeloid cells. CLRs are important for cell–cell communication and host defense against pathogens through the recognition of specific carbohydrate structures. Similar to a family of Toll-like receptors, CLRs signaling are involved in the various steps for initiation of innate immune responses and promote secretion of soluble factors such as cytokines and interferons. Moreover, CLRs contribute to endocytosis and antigen presentation, thereby fine-tune adaptive immune responses. In addition, there may also be a direct activation of acquired immunity. On the other hand, glycans, such as mannose structures, Lewis-type antigens, or GalNAc are components of tumor antigens and ligate CLRs, leading to immunoregulation. Therefore, agonists or antagonists of CLRs signaling are potential therapeutic reagents for cancer immunotherapy. We aim to overview the current knowledge of CLRs signaling and the application of their ligands on tumor-associating immune response.

## Introduction

Interaction between tumors and the immune system is a complex and dynamic process. The immune system consists of innate and adaptive immunity whose cooperative interactions are required for eliminating pathogens efficiently. Similar protective mechanisms are effective against cancer cells; the endogenous non-self which potentially grow into harmful cell mass. To prevent and suppress such tumor progression, the immune system utilize host defense mechanisms (1, 2).

Protecting self from harmful pathogens, and facilitating the symbiosis with harmless environmental microorganisms are the original mission of immune system. Above all, the innate immune system provides the first line of host defense against invading pathogens, with use of soluble factors, anti-microbial peptides, compliments, and natural antibodies. Initial activation of innate immune cells are mediated via pattern recognition receptors (PRRs) by recognizing characteristic structures of microorganisms (3, 4). Known PRRs are categorized into Toll-like receptors (TLRs), Nod-like receptors (NLRs), RIG-I-like receptors (RLRs), C-type lectin receptors (CLRs), and cyclic GMP–AMP synthase (cGAS) that has been recently identified.

Toll-like receptors and CLRs are involved in antigen capture, presentation, and activation of immune responses by enhancing cytokine/chemokine production and up-regulation of MHC class II molecules (5–7). NLRs predominantly recognize microbial products and endogenous danger signals, and enhance caspase activity to produce activated IL-1β (8). RLRs and cGAS are involved in cytosolic recognition of nucleic acids and other microbial components, i.e., RLRs are sensors of cytosolic dsRNA and cGAS are sensors of DNA, respectively, and both induce type I IFN production (9, 10).

**Figure 1:**
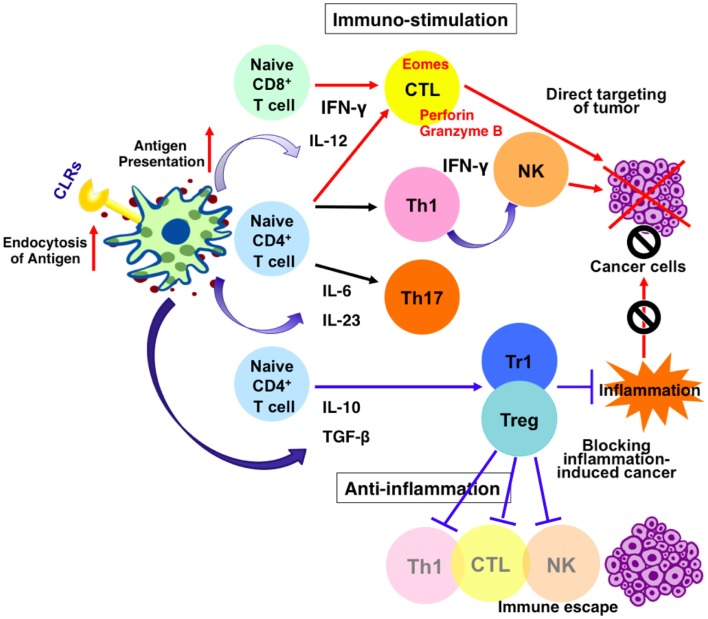
**Effects of CLRs signaling on dendritic cells and anti-cancer immune response**. Stimulation of CLRs enhances endocytosis of antigens and up-regulate antigen presentation. It also increases the production of mediators such as cytokines and interferons. Thus, CLRs–ligands possibly contribute to enhance anti-tumor immunity via two independent mechanisms. One mechanism leads to enhancement of tumoricidal activity of NK cells and cytotoxic T lymphocytes (CTL) via induction of IFN-γ and target cancer cells directly. The other mechanism support maturation of anti-inflammatory cells and lower the level of local inflammation, blocking inflammation-induced cancer.

C-type lectin receptors are a large family of receptors that encompass upwards of 1000 members with diverse functions including cell adhesion, complement activation, tissue remodeling, platelet activation, endocytosis, phagocytosis, and activation of innate immunity (11, 12). CLRs contain one or more C-type lectin-like domains, which are important for the recognition of specific carbohydrate structures of pathogens and self-antigens (13). Because of their specificity for glycans, such as mannose structures, Lewis-type antigens, or GalNAc (14, 15), CLRs may also mediate specific interactions with tumor antigens and facilitate tumor rejection. On the other hand, tumor cells devise multiple strategies to inhibit effector anti-tumor immune responses through modulating CLRs signaling (16, 17). It is therefore important to identify CLRs signaling toward immune evasion and regulate them in a specific way, while making the best application of beneficial side of CLRs signaling to mount anti-tumor immunity (Figure 1).

## The Immune Regulation by CLRs and Signaling Pathways

C-type lectin receptors are widely expressed on myeloid cells, such as macrophages, neutrophils, and dendritic cells (DCs). They contain one or more C-type lectin-like domains, which are important for recognition and internalization of glycosylated antigens. Ligand activation of CLRs initiates intracellular signaling pathways that regulate the immune response. Mounting evidence has been shown that CLRs play roles in sharping innate immune response. Many CLRs such as dectin-1, dectin-2, dectin-3, Mincle, and DEC-205 have been demonstrated to trigger cellular immune responses, including DC maturation, chemotaxis, reactive oxygen species production, and inflammasome activation (18, 19). The innate immune cells stimulated through CLRs acquire the capacity to secrete pro-inflammatory and anti-inflammatory cytokines such as TNF-α, IL-12, IL-6, IL-1β, and IL-10 (20–22). On the other hand, ligand engagement of some CLRs, such as MICL and DCIR, has inhibitory effects on host immunity through controlling DC maturation, activation, and proliferation (23–25).

**Figure 2:**
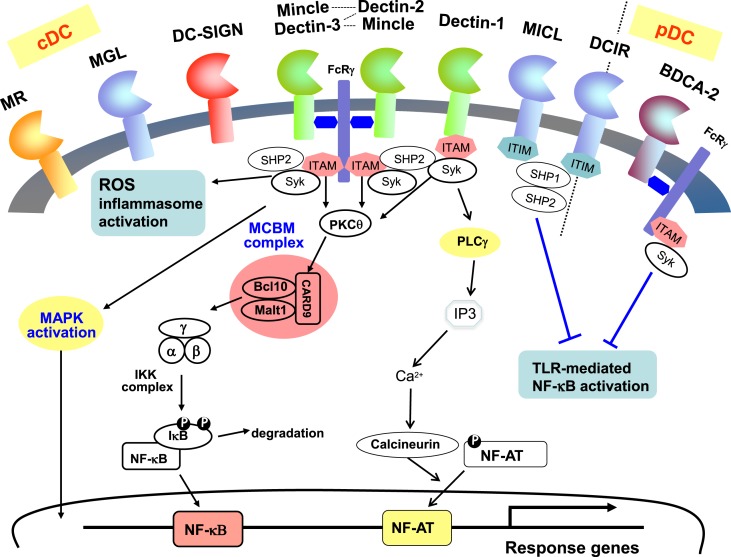
**Signaling pathways associated with CLRs on dendritic cells**. CLRs are dominantly expressed on myeloid cells such as dendritic cells and macrophages. MR, MGL, DC-SIGN, Mincle, Dectin-1, Dectin-2, MICL are expressed on cDCs, and BDCA-2 is expressed on pDCs, whereas DCIR is expressed on both cDCs and pDCs. Syk kinase/CARD9 pathway is activated by some CLRs signaling and mediates cell activation. ITAM-containing FcR are associated with Mincle, dectin-2, dectin-3 (MCL), and BDCA-2. Dectin-1 and DC-SIGN contain ITAM-like motifs whereas MICL and DCIR contain ITIM motifs in their cytoplasmic tails.

The ability of CLRs to exhibit activation or inhibition of immune response is regulated by the specific motifs in their cytoplasmic tails. Intracellular signaling through CLRs with immune-receptor tyrosine-based activation motif (ITAM) domains result in cell activation, whereas CLRs which possess immune-receptor tyrosine-based inhibition motif (ITIM) domains usually mediate inhibitory functions (18, 26). The tyrosine residues are phosphorylated by Src family kinases and a tri-molecular complex composed of CARD9, Bcl10, and MALT1 is involved in the subsequent activation of NF-κB and expression of inflammatory cytokines (6, 27, 28). Syk/CARD9 pathway is utilized by dectin-1, dectin-2, dectin-3, or Mincle and plays important roles in bridging the innate immunity and adaptive immunity. Dectin-1 directly signals through Syk using cytoplasmic ITAM and activates NF-κB, whereas dectin-2, dectin-2/dectin-3 heterodimer, and Mincle couple to Syk via the FcRγ and mediate NF-κB activation (29–32) (summarized and depicted in Figure 2). Signaling through Syk/IRF5 is crucial for the production of dectin-1-mediated IFN-β (33). Furthermore, it is reported that dectin-1 activates inflammasomes and caspase-1, leading to production of IL-1β (34).

Stimulation of these CLRs has been shown to drive the development of Th1, Th17, and CD8^+^ cytotoxic T lymphocytes (CTLs) cells immune responses through triggering the production of multiple cytokines (26, 35–37). In particular, dectin-1 has been found to activate NFAT also and enhance IL-2 and IL-10 production in DCs (38). A further study found that Src-homology phosphatase (SHP)-2 is an essential component, which facilitates the recruitment of Syk to the dectin-1 or the ITAM-containing adaptor FcRγ of dectin-2/3 and Mincle, and mediates the induction of Th17 responses (39). Given that T-cell immunity is essential for anti-tumor immunity, activation of ITAM-based CLRs signaling should support the development of protective immunity.

Recently, the important role of CLRs in inducing immunological tolerance has also been demonstrated. In the case of inhibitory CLRs containing ITIMs, such as DCIR (on dendritic cells) or MICL (on granulocytes and monocytes), SHP is an essential element. Ligation of these CLRs results in phosphorylation of ITIM domain, leading to SHP-1 and SHP-2 activation and inhibits cellular activation (25). Ligation of DCIR increases the number and function of Foxp3^+^ Treg cells, thus attenuates airway hyper responsiveness and inflammation (40). BDCA-2 and DC-SIGN do not contain a cytoplasmic ITIM motif but signaling through these CLRs has been shown to modulate TLR signaling through alternative pathways (41) and be critical for the maintenance of Foxp3^+^ Treg cells (42, 43). Moreover, several CLRs such as DC-ASGPR, SIGNR1, and dectin-1 are shown to play an important role in triggering IL-10-producing suppressive CD4^+^ T cells (44–47). Recently, it is highlighted that inflammation-induced cancers are prevented by anti-inflammatory mechanisms including Tregs (48). Therefore, the anti-inflammatory pathway lead by CLRs activation may also become a therapeutic strategy for reducing the risk of such diseases (Figure 1).

## Recognition of Tumor-Associated Antigen by CLRs

Tumors are recognized by the immune system through tumor antigens, including membrane proteins and altered carbohydrate molecules of glycoproteins or glycolipids on the cell surface (49). Tumor-associated carbohydrate antigens (TACAs) can be specifically recognized by CLRs. It has been shown that DC-SIGN recognizes carcinoembryonic antigen (CEA), a well-known tumor-associated antigen overexpressed on almost all human colorectal, gastric, and pancreatic adenocarcinomas, 70% of non-small cell lung carcinomas, and 50% of breast carcinomas A (50). DC-SIGN also exhibits high affinity for Mac-2-binding protein (Mac-2BP), which increases in patients with pancreatic, breast, and lung cancers (51).

Macrophage galactose type C-type lectin (MGL) is involved in the recognition and binding of tumor-associated Neu5Ac-Tn and Neu5Gc-Tn antigens (52). It has also been demonstrated that DCs are able to recognize cancer-specific glycosylation changes of the mucin 1 (MUC1), in particular, the carbohydrate sialyl Lewis X, and the sialyl TN epitope through MGL and DC-SIGN (53, 54). In addition, MUC1, CA-125, and TAG-72 show strong binding activity to mannose receptor (MR) and induce its internalization (55–57). Further, mannose-binding lectin (MBL) has been shown to recognize glycoproteins from a human colorectal carcinoma cell line in a fucose-dependent manner (58–60).

A critical role of dectin-1, a receptor for β-glucans (61, 62), has recently been shown in recognition of N-glycan structures on tumor cells. N-glycosidase treatment markedly reduced the binding of dectin-1 to tumor cells. Importantly, tumoricidal activity of splenocytes was reduced when tumor cells were pretreated with N-glycosidase (63).

**Figure 3:**
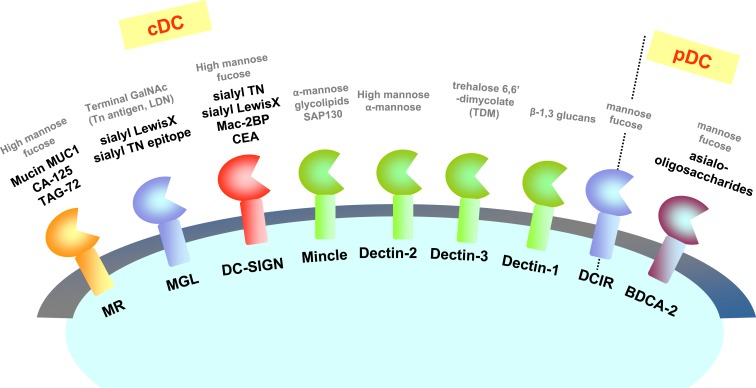
**CLRs and their ligands on tumor cells**. CLRs recognize carbohydrate structures including tumor antigens. Known ligands expressed on tumor cells are represented with bold black letters. Known ligands other than tumor cells (such as yeasts) are represented with gray letters as references.

Plasmacytoid dendritic cells (pDCs) are responsible for production of type I interferons (IFN-α and β), type III IFNs (IFN-λ/IL-28/29), and pro-inflammatory cytokines. Antigen presentation by CpG-activated pDC influenced anti-tumor immune responses by promoting efficient Th17 differentiation (64). A study showed that BDCA-2 exclusively expressed on pDCs binds tumor cells via asialo-oligosaccharides containing terminal residues of galactose (65) and potently suppresses the ability of pDCs to produce type I IFNs. Such direct regulation and/or cross-regulation of TLRs signaling by BDCA-2, an inhibitory CLR, may also suppress beneficial adaptive immune response *in vivo* (Figure 3).

## CLRs in Induction of Anti-Tumor Immune Response

Effective immunological eradication of tumors requires NK cells and tumor-specific CD8^+^ and CD4^+^ T cells. The potential role of CLRs improving anti-tumor activity of immune cells has been investigated. A study showed that MGL interacts with tumor-associated Tn antigens and efficiently internalized with antigens for presentation to CD4^+^ T cells (5). Furthermore, engagement of MGL using α-*N*-acetylgalactosamine-carrying tumor-associated antigens promotes the up-regulation of maturation markers of DCs, decrease phagocytosis, enhance motility, and most importantly increase antigen-specific CD8^+^ T-cell activation (54).

DC-SIGN is another important CLR in inducing anti-tumor immune responses. It is reported that Lewis X oligosaccharides–heparanase complex activate and enhance the maturation of DCs, leading to enhancement of antigen-specific IFN-γ production and cytotoxic T-cell response. Furthermore, the modified DCs also significantly suppress the established tumor growth and prolong the life span of tumor-bearing mice (66). In addition, glycan-modified liposomes lead to efficient antigen presentation of DCs in the presence of LPS and augment CD4^+^ and CD8^+^ effector T-cell activation via DC-SIGN-dependent pathway (67). The potency of MR to improve anti-tumor immune responses has also been conducted. Cross-presentation of antigen and strong antigen-specific immune response were induced by conjugation of glycan ligands to MR (68), which resulted in an efficient anti-tumor response and tumor clearance (69).

Dectin-1 is one of the most important CLRs and its contribution to anti-tumor immunity has been intensively studied. Dectin-1 engagement is apparent to up-regulate costimulatory molecules such as CD80, produce TNF-α, IL-6, IL-2, IL-10, IL-12, and IL-23, and elicit potent CTL responses that protect mice from tumor challenge (35). Targeting of dectin-1 with its ligands β-glucan has been shown to increase the infiltration of activated T cells into the tumor. On the other hand, the number of tumor-caused immunosuppressive regulatory T cells and myeloid-derived suppressor cells are decreased (70, 71). More recently, the critical role of dectin-1 on enhancement of NK-mediated killing of tumor cells has been demonstrated. Dectin-1 recognize N-glycan structures on the surface of some tumor cells, and cause the activation of IRF5 transcription factor and downstream gene induction, for the full-blown tumoricidal activity of NK cells (63).

As described above, MR and DC-SIGN are major players for both immune evasion and eradication of tumor cells. Further information is necessary to clarify how these CLRs signaling affect the direction of the immunological outcome. Whether cell types or expression level is important, or ligands and microenvironment is the key, or maybe both are closely related. It is known the nature of ligands (i.e., size, form, or chemical side chains of ligands) directly modulate CLRs signaling (62). Further investigation on such regulation of CLRs signaling should lead to make the best application of beneficial side of CLRs signaling to mount anti-tumor immunity.

## CLRs and Tumor Immune Evasion

C-type lectin receptors mediate beneficial effect on anti-tumor immunity via enhancement of type I and type II interferon production. On the other hand, CLRs signaling also play roles on induction of anti-inflammatory factors and molecules (23), and suppress TLRs-mediated protective immunity, thereby tolerating cancer cells escape from immune surveillance. Some examples of such process are induction of specific tolerance to tumor antigens, TGF-β and/or IL-10 production, down-regulation of MHC molecules, or up-regulation of FasL expression (72). Several studies have shown the involvement of CLRs on dysfunction of anti-tumor immune responses. The interaction between DC-SIGN and tumor-associated Le glycans results in enhanced IL-10 production, and impairs production of pro-inflammatory cytokines in tumor-associated macrophages (TAMs) from breast adenocarcinoma and melanoma patients, which leads to decrease capacity to elicit anti-tumor T-cell responses (73). Ligation of DC-SIGN and tumor-associated Le glycans also strongly enhance LPS-induced anti-inflammatory cytokine secretions of IL-6 and IL-10 by monocyte-derived DCs (50). Therefore, ligation of DC-SIGN might cause tumor progression by contributing to the maintenance of an immunosuppressive environment.

Other CLR associated with tumor immune evasion is MR. The research study showed that tumor-activated liver sinusoidal endothelial cells (LSECs) affect liver sinusoidal lymphocytes (LSLs) anti-tumor cytotoxicity and IFN-γ/IL-10 secretion through MR-dependent mechanisms. Further, immunosuppressive effects of tumor-activated LSECs on LSLs were abrogated by way of anti-mouse MR antibodies or MR^−/−^ mice (74).

Recently, the important role of CLRs on modulating the function of tumor-associated cells in tumor microenvironment has been demonstrated. TAMs are a major component of the tumor stroma, which contribute to the evasion of tumors from immune control by producing immune-suppressive cytokines such as IL-10 and TGF-β (75). It has been found that TAMs from human ovarian carcinoma abundantly express MR and dectin-1, MDL-1, MGL, DCIR. MR engagement by tumoral mucins and an agonist anti-MR antibody modulates cytokine production by TAMs toward an immune-suppressive profile: increase of IL-10, absence of IL-12, and decrease of the Th1-attracting chemokine CCL3, indicating that tumoral mucin-mediated activation of the MR on TAMs is important for their immune-suppressive phenotype (57).

In addition to expressing in immune cells, some CLRs have been shown to express on tumor cells, and involved in suppressing human immune system function. LSECtin, a cell-surface member of the C-type lectin DC-SIGN, has been found to express in B16 melanoma cells and inhibit tumor-specific T-cell responses (76). It is therefore important to identify such self-recognition toward immune evasion and regulate them in a specific way.

## Genetic Variation of CLRs and Cancers

Host genetic background is one of important factors influencing susceptibility to cancer. Recently, study on single nucleotide polymorphisms (SNP) has been widely used to explore genetic susceptibility. SNPs in CLRs loci have been investigated to clarify its relationship to inflammatory responses. Because chronic inflammation is highly associated with the onset and progression of a multiplicity of human cancer, it is possible SNPs in CLRs associate with cancer susceptibility. Lu et al. (77) evaluated the correlation between colorectal cancer (CRC) risk and SNPs in three C-type lectin genes, i.e., DC-SIGN, MBL, and REG4. They found that polymorphisms in DC-SIGN gene promoter were associated with increased risk in CRC patients, while a SNP in REG4 might be a useful marker for CRC progression. The association of polymorphisms of genes encoding DC-SIGN with nasopharyngeal carcinoma risk has also been investigated. Three SNPs in the GG genotype of the rs2287886, AA genotype of the −939 promoter polymorphism, and the G allele of the rs735239 are connected with increased risk of nasopharyngeal carcinoma (78).

Mannose-binding lectin, soluble CLRs, is a plasma collectin and one of the key molecules involved in modulating innate immune system. Low level of serum MBL is associated with increased risk of colon cancer. Polymorphisms in the 3′-untranslated region of MBL2 at rs10082466, rs2120132, rs2099902, and rs10450310 reduce MBL plasma levels and activity (79). Odds ratio for homozygous variants versus wild-type ranged from 3.17 to 4.51, whereas the 3′-UTR region haplotype consisting of these four variants had an OR of 2.10.

## Ligand Treatment or Blockade of CLRs and Cancer

Based on the immune-regulatory effects of CLRs on cellular immunity, application of their ligands to cancer therapy is a scheme of promising scope. Several CLR agonists or antagonists are candidates for anti-cancer drugs. β-glucan as dectin-1 agonists has been extensively investigated for their anti-tumor activity. In murine lung carcinoma models, orally administered particulate β-glucans significantly inhibited tumor growth (71, 80). Both oral and intraperitoneal injection of highly purified soluble β-glucan derived from *Grifola frondosa* were reported to exert anti-tumor effects in experimental murine mammary and colon adenocarcinoma tumor models (70, 81). In addition to their direct effects on specific immunity, β-glucans significantly augment the therapeutic efficacy mediated by anti-tumor monoclonal antibodies (mAbs) in murine breast, liver metastasis, lung, and lymphoma tumor models as well as in human neuroblastoma, lymphoma, and melanoma xenograft models (82). In human, the combination therapy of β-glucan and conventional chemotherapy was reported to improve the long-term survival of patients with ovarian cancer (83). A meta-analysis shows that the addition of lentinan (a purified β-glucans isolated from shiitake mushroom) to chemotherapy prolonged the survival of patients with advanced gastric cancer as compared to chemotherapy alone (84).

Some mechanisms have been proposed to explain the therapeutic response of β-glucan on anti-tumor activity. First, β-glucans are capable of eliciting anti-tumor innate and adaptive immune response via dectin-1-dependent pathway. As discussed above, β-glucans play an essential role in activating DCs and macrophages both *in vitro* and *in vivo*, leading to enhanced antigen-specific CD4^+^ and CD8^+^ T-cell responses. Moreover, β-glucans modulate the suppressive tumor microenvironment and facilitate anti-tumoral cellular immunity.

The other important role of CLRs is to serve as sensors that transduce tumor antigen into DCs. Some CLRs, including MGL, MR, DNGR-1, and DEC-205, have been found to deliver exogenous antigens on MHC-I for inducing efficient CTL immune response and MHC-II for stimulation of CD4^+^ T cells (68, 85, 86). Moreover, targeted delivery of tumor antigens via DC-SIGN, DNGR-1, and DEC-205 with an appropriate adjuvant is capable to prevent development or mediate eradication of tumor in grafted mouse models (87–90).

Along with the rapid and thorough innate immune systems, targeting CLRs has emerged as a translational approach to treat a wide variety of cancers. However, there still are some problems yet resolved and further research is required for improving the anti-tumor strategies via CLRs. Some CLRs signaling results in immunosuppressive responses, for instance, and lead to tumor immune escape. Drugs targeting immune checkpoint molecules such as PD-1, PD-L1, and CTLA-4 have recently been demonstrated beneficial and safe (91, 92). The combination of strategy targeting CLRs and immune checkpoints may improve anti-tumor effectiveness.

## Concluding Remarks

C-type lectin receptors are multifunctional receptors that have a key role in the recognition of pathogens and regulating innate and adaptive immune responses. In fact, abundant evidence supports that CLRs, especially on DCs, contribute to the recognition of TACA. CLRs also play important roles in inducing anti-tumor immune response and regulate tumor-promoting inflammation. On the other hand, the function of CLRs in tumor remains unknown, therefore CLRs may act as double-edged swords in tumor-associated immune response. Specific regulation of CLRs signaling by modulating tumor microenvironment such as glycoligands and immune cells should lead to the best application of CLRs biology.

## Conflict of Interest Statement

The authors declare that the research was conducted in the absence of any commercial or financial relationships that could be construed as a potential conflict of interest.
